# Metformin dual-targets metabolism and survival pathways in BPDCN

**DOI:** 10.1016/j.isci.2026.116323

**Published:** 2026-06-13

**Authors:** Zineb Mekkaoui, Ludivine Dal Zuffo, Mathieu Vetter, Maxime Fredon, Margaux Poussard, Sabeha Biichle, Virginie Mougey, Patricia Mercier-Letondal, Gwenaël Rolin, Yann Godet, Sylvain Perruche, Francine Garnache-Ottou, Philippe Saas, Mourad Aribi

**Affiliations:** 1 Laboratory of Applied Molecular Biology and Immunology, W0414100, University of Tlemcen, Tlemcen 13000, Algeria; 2 Université Marie et Louis Pasteur, EFS, INSERM, RIGHT (UMR 1098), 25000 Besançon, France; 3 CARLA Biotherapeutics, 25000 Besançon, France; 4 Université Marie et Louis Pasteur, CHU Besançon, EFS, INSERM, RIGHT (UMR 1098), 25000 Besançon, France; 5 DImaCell Imaging Resource Center, Université Marie et Louis Pasteur, 25000 Besançon, France; 6 MED'INN'Pharma, 25000 Besançon, France; 7 Institute for Advanced Biosciences, Team: Cell Dynamics, Immunity, Metabolism, & Cancer, Inserm U1209, CNRS UMR5309, University Grenoble Alpes, 38000 Grenoble, France; 8 Etablissement Français Du Sang Auvergne-Rhône-Alpes, R&D Laboratory, 38000 Grenoble, France; 9 China-Algeria International Joint Laboratory on Emergency Medicine and Immunology, Guangdong Provincial, People’s Hospital (Guangdong Academy of Medical Sciences), Southern Medical University, Guangzhou, China; 10 Algerian Society for Experimental and Applied Immunology (ASEAI), Tlemcen, Algeria

**Keywords:** health sciences, therapeutics, biological sciences, medical biochemistry, cancer

## Abstract

Blastic plasmacytoid dendritic cell neoplasm (BPDCN) is a rare and aggressive hematologic malignancy with limited therapeutic options. Metformin, a commonly prescribed antidiabetic drug, has recently gained attention for its anticancer potential, but its effects on BPDCN remain unknown. Here, we show that metformin reduces cell viability and induces caspase-dependent apoptosis in both established (CAL-1, GEN2.2) and primary BPDCN cells, partly through activation of the intrinsic apoptotic pathway. Mechanistically, metformin activates AMPK and disrupts mitochondrial respiration and glycolysis, while inhibiting key oncogenic signaling pathways including Akt/mTOR, NF-κB, STAT3, and STAT5. *In vivo*, metformin reduces tumor cell infiltration in the spleen and modulates NF-κB and STAT5 signaling, although its effect on overall disease progression is limited. These results identify metformin as a multifaceted agent targeting both metabolic and survival pathways in BPDCN, supporting its potential as a therapeutic strategy in this rare malignancy.

## Highlights

• Metformin reduces BPDCN cell viability *via* an AMPK-independent mechanism
• Metformin disrupts mitochondrial respiration and glycolysis
• Metformin inhibits Akt, mTOR, NF-kB p65, STAT3, and STAT5 signaling
• Metformin targets key survival and metabolic pathways in BPDCN cells

## Graphical abstract

**Figure:**
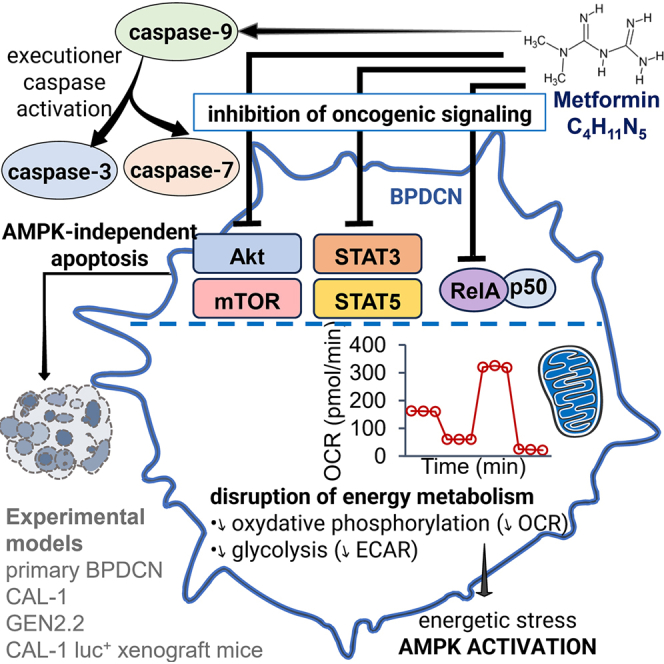

## Introduction

Blastic plasmacytoid dendritic cell neoplasm (BPDCN) is a rare malignancy derived from pDCs associated with a poor prognosis. It was classified in the 2022 World Health Organization classification of haematolymphoid tumors under histiocytic/dendritic cell neoplasms.^1^

Given the limited therapeutic options and poor prognosis associated with BPDCN, there is an urgent need to explore novel treatment strategies. One promising candidate is metformin, a biguanide derived from French lilac (*Galega officinalis*), which is widely used for the treatment of type 2 diabetes.^2^ Metformin has been shown to inhibit cancer cell viability, growth, and proliferation.^3^^,^^4^^,^^5^ Previous studies conducted on cancer cell lines and murine tumor models have demonstrated its potential antileukemic activity,^6^^,^^7^ mediated through both AMP-activated protein kinase (AMPK)-dependent^3^^,^^6^ and AMPK-independent mechanisms.^7^^,^^8^ Metformin is well-known to activate AMPK.^2^^,^^3^^,^^9^ AMPK, a key regulator of cellular energy homeostasis, is activated by metabolic stresses that either inhibit mitochondrial ATP production or accelerate ATP consumption. AMPK activation triggers several pathways (e.g., glycolysis) to generate energy.^3^^,^^6^

Constitutive activation of different signaling pathways such as nuclear factor-κB (NF-κB) p65 (p50/p65/RelA),^10^^,^^11^ Akt/mammalian target of rapamycin (mTOR),^12^ signal transducer and activator of transcription 3 (STAT3),^13^ and STAT5^7^ has been identified as hallmarks of BPDCN. Previous studies have shown that metformin downregulates NF-κB p65,^10^^,^^14^ Akt/mTOR^13^; STAT3/STAT5^7^ activation in both solid and hematological tumors.

These findings suggest that metformin can inhibit cell proliferation, survival, and energetic metabolism. Although metformin has been proposed to exhibit selectivity for hematological cells,^15^ its effects on BPDCN remain unexplored. In this study, we investigated the mechanisms underlying metformin-induced cell death in BPDCN.

## Results

### Metformin inhibits BPDCN cell proliferation and cell viability in a concentration-dependent manner

**Figure 1:**
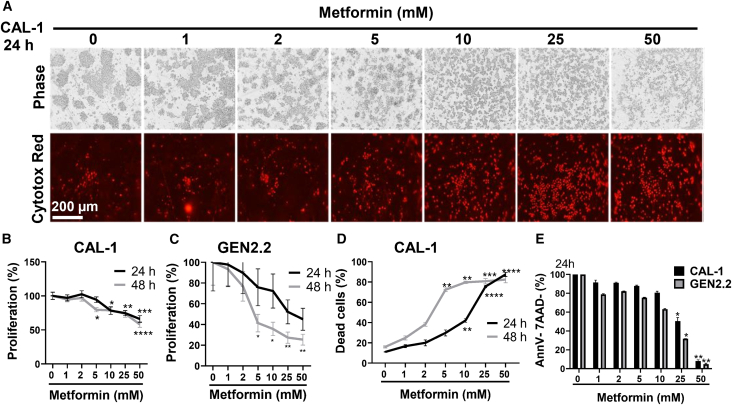
Metformin inhibits cell proliferation and survival of blastic plasmacytoid dendritic cell neoplasm (BPDCN) cell lines CAL-1 and GEN2.2 BPDCN cells were treated with increasing concentrations of metformin (0–50 mM) for 24 or 48 h. Cell proliferation (A–C) and cell death (A and D) were analyzed using the IncuCyte S3® live-cell analyzer. (A) CAL-1 cells 24 h after treatment were shown (10 X image magnification) with upper images (phase) representing phase-contrast imaging while lower images (Cytotox Red) show fluorescence imaging after staining with cytotox red dye, which allows to count dead cells (labeled in red). Scale bars, 200 μm. (B and C) Metformin inhibits proliferation of CAL-1 and GEN2.2 cells in a concentration-dependent manner. (D) Metformin increases cell death of CAL-1 cells in a concentration-dependent manner. Data are expressed as the mean ± SEM of 8 independent wells per condition. (E) Viable cells were defined by flow cytometry as Annexin-V^–^/7-AAD^–^ CAL-1 (black) or GEN2.2 (gray) cell lines after treatment with metformin (0–50 mM) for 24 h. Untreated CAL-1 and GEN2.2 cells were arbitrarily assigned to a 100%. Histograms represent the mean ± SEM of three independent experiments. *p* value is calculated using one-way ANOVA (more than two groups). ∗*p* < 0.05, ∗∗*p* < 0.01, ∗∗∗*p* < 0.001, ∗∗∗∗*p* < 0.0001.

To evaluate the effects of metformin on BPDCN cells, we treated CAL-1 and GEN2.2 cells with increasing concentrations of metformin (0–50 mM) for 24 h or 48 h. Cell proliferation was monitored every 2 h over a 48-h period using the IncuCyte live-cell imaging system, considering cell confluence as the primary parameter. Phase-contrast imaging further demonstrated that metformin treatment led to a reduction in cell clump formation (Figure 1A). Proliferation curves generated from these data revealed that metformin inhibited in a concentration-dependent manner cell viability and proliferation in both CAL-1 (*n* = 2, 8 wells/condition) and GEN2.2 (*n* = 1, 8 wells/condition) cells, (*p* < 0.01, after 48 h treatment with at 5 mM metformin) (Figures 1B, 1C, and 1D).

To further characterize the sensitivity of BPDCN cell lines to metformin, we incubated CAL-1 cells with metformin (0–50 mM) for 48 h in the presence of cytotox red dye (*n* = 1, 8 wells/condition), an exclusion dye which labels dead cells. Real-time monitoring using the IncuCyte live-cell analyzer revealed that untreated CAL-1 cells exhibited a baseline cell death rate of 11.0% ± 1.3%. In contrast, metformin-treated cells showed a concentration-dependent increase in cell death, reaching 87.5% ± 2.0% at 50 mM (*p* < 0.0001) after 24 h (Figures 1A and 1D).

These findings were corroborated by flow cytometry analysis of Annexin-V/7AAD-stained cells, which confirmed that metformin treatment significantly induced cell death in both CAL-1 and GEN2.2 cells (*n* = 3, Figure 1E) as well as in BPDCN cells isolated from three patient-derived xenografts (PDX) (Figure S1A) in a concentration-dependent manner with an IC50 value of 36 mM and 35.6 mM after 24 h treatment in CAL-1 and GEN2.2 cells, respectively (Figures S1B and S1C). In contrast, peripheral blood mononuclear cells (PBMCs) from 3 healthy donors exhibited an IC50 of approximately 56 Mm (Figures S1D and S1E). Accordingly, compared to CAL-1 and GEN2.2, PBMCs demonstrated reduced sensitivity, as indicated by a rightward shift of the dose-response curve.

### Metformin induces apoptosis in human BDPCN cell

**Figure 2:**
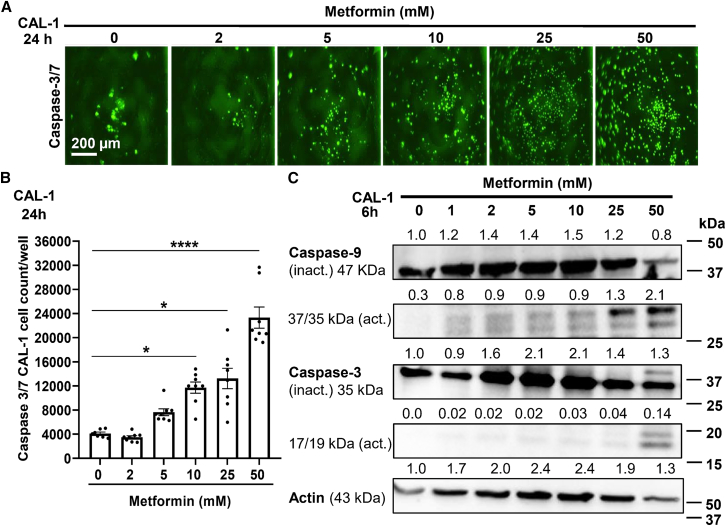
Metformin induces apoptosis in blastic plasmacytoid dendritic cell neoplasm cell line CAL-1 cells were treated with increasing concentration of metformin (0–50 mM) in the presence of IncuCyte® caspase-3/7 Dye for 48 h and were imaged at 10 X magnification in the IncuCyte S3® live-cell analyzer every 2 h. (A) Representative image of caspase-3/7 green fluorescence emitted by CAL-1 cells 24 h after treatment. Scale bars, 200 μm. (B) Histogram represents the mean ± SEM of caspase-3/7 green fluorescent positive CAL-1 cells per well (8 independent wells per condition) and statistically significant differences are indicated. (C) CAL-1 cells were treated, 6 h with increasing concentration of metformin (0–50 mM), and expression of caspase-3, cleaved caspase-3, caspase-9, and cleaved caspase-9 were evaluated by western blot analysis and semi-quantified. One representative experiment of two is shown. The expression of proteins was normalized with actin and non-treated condition. Actin was used as control for protein expression. *p* value is calculated using one-way ANOVA (more than two groups). ∗*p* < 0.05, ∗∗*p* < 0.01, ∗∗∗*p* < 0.001.

To determine the cell death mechanism induced by metformin in BPDCN cells, we treated CAL-1 cells with increased concentrations of metformin (0–50 mM) for 24 h or 48 h in the presence of the IncuCyte caspase-3/7 green dye (*n* = 1, 8 wells/condition), which specifically labels executioner caspases, and thus apoptotic cells. Real-time analysis using the IncuCyte live-cell analyzer revealed a concentration-dependent increase in green fluorescent-positive apoptotic cells (mean ± SEM: 11715 ± 929 *vs*. 4101 ± 244 green fluorescent positive cells/well in metformin (10 mM)-treated *vs*. untreated cells, respectively, *p* < 0.05) (Figures 2A and 2B). These findings strongly suggested that metformin induced apoptosis in BPDCN cells.

To further validate these results, we performed western blot analysis on CAL-1 cells treated with increased concentrations of metformin (*n* = 2, 0 to 50 mM) for 6 h. This investigation revealed cleavage of both caspase-3 and caspase-9, an executioner caspase and an initiator caspase of the intrinsic apoptotic pathway, respectively. This confirms that metformin triggers caspase-dependent apoptosis, at least through the intrinsic pathway (Figure 2C).

### Metformin-mediated cell death in BPDCN cells is independent on AMPK activation

**Figure 3:**
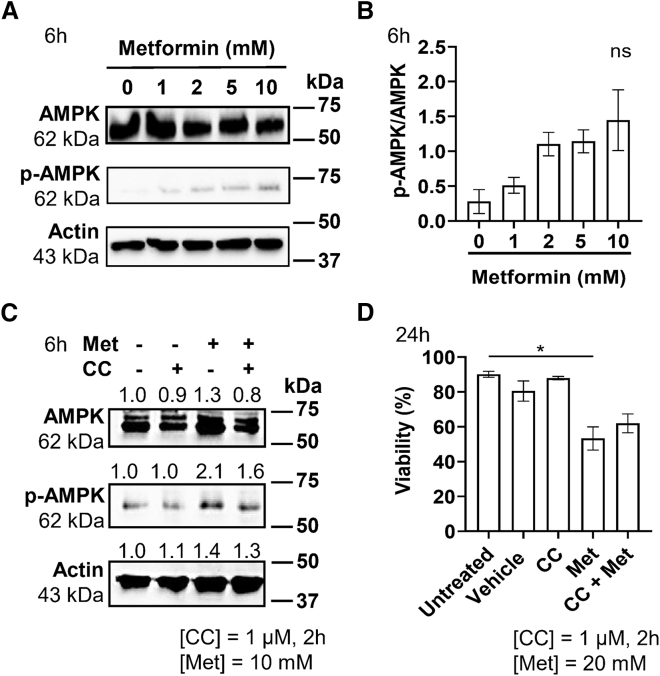
Metformin activates AMPK signaling pathway in blastic plasmacytoid dendritic cell neoplasm cell line CAL-1 (A) Expression levels of AMPK and phosphorylated AMPK (*p*-AMPK) in CAL-1 cell line were analyzed by western blotting 6 h after treatment with increasing concentrations of metformin (0–10 mM). One representative experiment out of three is shown. (B) Expression was semi-quantified in CAL-1 cells and normalized to AMPK and, relative to the untreated condition. Actin was used as a loading control. Data are presented as mean ± SEM of the *p*-AMPK to AMPK ratio from three independent experiments. (C) CAL-1 cells were pretreated or not with 1 μM of AMPK inhibitor CC for 2 h, and then treated or not with 10 mM metformin for 6 h. (D) Cell viability of CAL-1 cells was assessed 24 h after treatment with 20 mM metformin in the presence or absence of 1 μM of AMPK inhibitor CC. Histogram represents the mean ± SEM of three independent experiments. The vehicle corresponds to DMSO used for CC reconstitution. Met, metformin; CC, compound C; ns, not significant. *p* value is calculated using one-way ANOVA (more than two groups). ∗*p* < 0.05.

To elucidate the mechanism by which metformin induced cell death in BPDCN cells, we focused on its effects on AMPK activation. This was addressed using two complementary approaches: one tested the ability of metformin to activate AMPK, and the other employed the specific AMPK inhibitor compound C (CC) to block AMPK activation in CAL-1 cells. As an initial step, CAL-1 cells were treated with increasing concentrations of metformin (0–10 mM) for 6 h (*n* = 3). Western blotting analysis revealed that metformin stimulated the phosphorylation of AMPK in CAL-1 cells in a concentration-dependent manner (Figure 3A) and as the ratio of p-AMPK to total AMPK was elevated (Figure 3B). This finding indicated that metformin activates the AMPK pathway in CAL-1 cells (Figure 2). Similar results were obtained with GEN2.2 cells (Figures S2A and S2B); Although basal AMPK phosphorylation was observed in GEN2.2 cells, a strong induction was noticed at the highest concentration (10 mM). Thus, metformin induces AMPK phosphorylation in BPDCN cell lines.

Building on these findings, we next sought to investigate whether AMPK activation plays a critical role in metformin-induced apoptosis. To do this, CAL-1 cells were pre-treated with or without 1 μM of CC for 2 h, followed by treatment with or without 10- or 20-mM metformin for 6 or 24 h (*n* = 3). Cell viability after 24 h of treatment with a cytotoxic concentration of metformin (20 mM) and the expression levels of p-AMPK after 6 h of treatment with less cytotoxic concentration (10 mM) were assessed by flow cytometry and western blotting, respectively. CC pretreatment decreased slightly the effect of metformin on AMPK phosphorylation compared to metformin treatment alone (Figure 3C). Additionally, as illustrated in Figure 3D, reduction in cell viability was still observed after pretreatment with CC before metformin addition. These results suggest that metformin activates the AMPK signaling pathway in BPDCN cells, but cell death induction remains mainly APMK-independent.

### Metformin interferes with the Akt/mTOR signaling pathway in BPDCN cells

**Figure 4:**
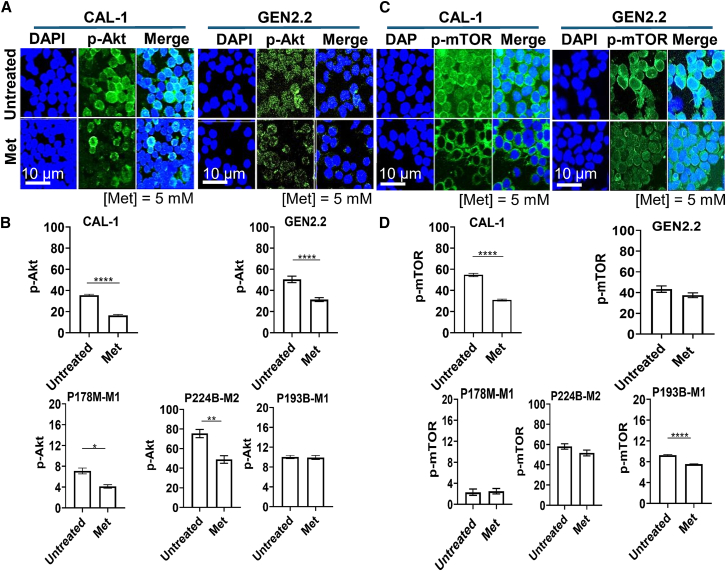
Metformin inhibits Akt/mTOR signaling pathway in blastic plasmacytoid dendritic cell neoplasm cell lines and patient-derived xenograft cells Three BPDCN cell models: CAL-1 (*n* = 3) and GEN2.2 (*n* = 3) cell lines as well as three patient-derived xenograft cells (PDX: P224B-M2 [*n* = 1], P178M-M1 [*n* = 1] and P193B-M1 [*n* = 1]) were treated 6 h with or without 5 mM metformin. (A) and (C) one representative experiment out of 3 showing intracellular expression of p-Akt and p-mTOR evaluated by confocal microscopy in CAL-1 and GEN2.2 cells (*n* = 3). Scale bars, 10 μm. (B) and (D) Protein expression levels in the CAL-1 and GEN2.2 cell lines and PDX cells were quantified with Zen Blue software. Histograms represent the mean ± SEM and statistically significant differences are indicated. *p* value is calculated using the Mann-Whitney *U* test (two groups). ∗*p* < 0.05, ∗∗*p* < 0.01, ∗∗∗*p* < 0.0001. Met: metformin. PDX: patient-derived xenografts.

Previous studies have shown that the Akt/mTOR signaling pathway plays a critical role in promoting cell proliferation in several cancers.^16^^,^^17^ In the present study, CAL-1 and GEN2.2 cells and three different BPDCN PDX were treated with or without 5 mM (a concentration associated with reduced cell proliferation, see Figures 1B and 1C) of metformin for 6 h. Confocal microscopy was then performed to assess the expression levels of *p*-Akt and *p*-mTOR in BPDCN cells. Compared with the control group (untreated), 5 mM of metformin significantly reduced phosphorylation levels of Akt (*n* = 3, *p* < 0.0001) in CAL-1 also inhibited *p*-Akt (*p* < 0.0001) in GEN2.2 cells and in two BPDCN PDX out of the three tested (Figures 4A and 4B). Metformin (5 mM) inhibited also *p*-mTOR in CAL-1 cells (*n* = 3, *p* < 0.0001) and was only observed in one BPDCN PDX out of the three tested (Figures 4C and 4D). These results suggest that metformin reduces the proliferative capacity of BPDCN cells, at least in part, through the inhibition of the Akt/mTOR signaling pathway.

To further delineate the involvement of mTOR signaling, we treated CAL-1 and GEN2.2 cells with the dual mTORC1/2 inhibitor PP242 alone or in combination with metformin. As shown in Figure S3, PP242 alone affected differently BPDCN cell lines with a significant effect on GEN2.2 cells but not CAL-1 cells. The combination with metformin (at non cytotoxic concentration) did not produce additive or synergistic effects, suggesting that metformin and PP242 may converge on the same mTOR-dependent survival pathway. Overall, these data suggest that metformin exerts its anti-tumor activity primarily through suppression of the mTOR axis, independently of AMPK.

### Metformin interferes with the phosphorylation of NF-κB p65 and inhibits STAT3 and STAT5 survival pathways in BPDCN cells

**Figure 5:**
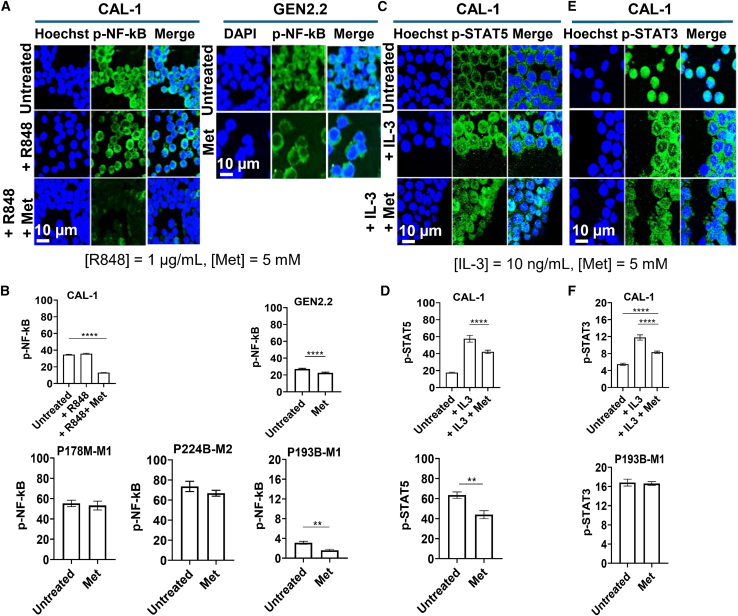
Metformin inhibits the NF-κB p65, STAT3, and STAT5 signaling pathway in blastic plasmacytoid dendritic cell neoplasm cell lines and patient-derived xenografts The intracellular expression levels of p-NF-κB p65, p-STAT3, and p-STAT5 were assessed using confocal microscopy in CAL-1 and GEN2.2 BPDCN cell lines and three different PDX cells incubated with or without 5 mM of metformin for 24 h. (A) A representative experiment out of three showing nucleus expression of p-NF-κB p65 in CAL-1 and GEN2.2 cells. CAL-1 cells were stimulated with TLR7 (using R848, 1 μg/mL) for 6 h to increase NF-κB activation. Scale bars, 10 μm. (B) Histograms represent p-NF-kB p65 expression in CAL-1 and GEN2.2 cell lines as well as in three different PDX cells (P224B-M2 [*n* = 1], P178M-M1 [*n* = 1] and P193B-M1 [*n* = 1]), represented as mean ± SEM. GEN2.2 and PDX cells were incubated with or without 5 mM metformin for 6 h without TLR7 stimulation to assess the effect on basal p-NF-κB expression. (C) and (E) CAL-1 cells were treated or without 5 mM metformin for 24 h followed by stimulation with 10 ng/mL IL-3 for 30 min. A representative experiment out of three showing intracellular expression of p-STAT5 (C) and p-STAT3 (E) in CAL-1 cells. (D) Cumulative data from three independent experiments quantifying p-STAT5 fluorescent intensity in CAL-1 cells and data from one experiment quantifying p-STAT5 fluorescent intensity in one PDX cell (P193B-M1) are shown as mean ± SEM. (F) p-STAT3 expression levels in CAL-1 cells (*n* = 3) and one PDX cell (P193B-M1, *n* = 1) were quantified, and cumulative data from three independent experiments in CAL-1 and from one experiment in PDX cells (P193B-M1) are represented as mean ± SEM. PDX cells were incubated with or without 5 mM metformin for 6 h in the absence of IL-3 stimulation. *p* value is calculated using the Mann-Whitney *U* test (two groups). ∗∗*p* < 0.01, ∗∗∗∗*p* < 0.0001. Met: metformin. PDX: patient derived xenografts.

As metformin displays various activities on a wide range of cell types, we investigated whether it interfered with NF-κB p65 phosphorylation in BPDCN cells, as NF-κB is recognized to be involved in survival signaling pathways^18^^,^^19^ and to be constitutively activated in BPDCN.^10^^,^^11^ CAL-1, GEN2.2, and BPDCN PDX were treated with a non-cytotoxic concentration of metformin (5 mM for 6 h, *n* = 3), and confocal microscopy was performed to assess the nuclear localization of phosphorylated NF-κB p65 (p-NF-κB p65). Metformin treatment significantly reduced the nuclear translocation of p-NF-κB p65, consistent with inhibition of NF-κB p65 activation. In CAL-1 cells stimulated with R848, the percentage of cells with nuclear p-NF-κB p65 decreased from 35.8% ± 0.5% to 13.0% ± 0.1% (*n* = 3, *p* < 0.0001, Figures 5A and 5B). Similarly, in GEN2.2 and one PDX BPDCN cells out of the three tested, metformin decreased NF-κB p65 phosphorylation (Figure 5B). These results suggest that NF-κB p65 inhibition by metformin contributes to its anti-proliferative effects in BPDCN cells.

Next, we aimed to determine whether metformin could disrupt two critical survival and proliferation pathways in BPDCN cells (*i*.*e*., STAT3 and STAT5 activation), which are often implicated in cancer progression and treatment resistance.^20^^,^^21^ CAL-1 cells were exposed to a non-cytotoxic concentration of metformin (5 mM for 24 h) and subsequently stimulated with IL-3 (10 ng/mL for 30 min),^11^ while one BPDCN PDX was treated with metformin for 6 h without IL-3 stimulation. Confocal microscopy analysis demonstrated a significant downregulation in the phosphorylation levels of STAT5 (Figures 5C and 5D) and STAT3 (Figures 5E and 5F) following CAL-1 cell treatment with metformin (*n* = 3, *p* < 0.0001). These findings demonstrate that metformin inhibited IL-3-induced activation of STAT3 and STAT5 in CAL-1 cells. Metformin also inhibited p-STAT5 (but not p-STAT3) in one BPBCN PDX. This may contribute to cytotoxic/anti-proliferative effects of metformin in BPDCN cells.

### Metformin inhibits mitochondrial respiration and reprograms metabolic pathways in BPDCN cells

To further elucidate the metabolic effects of metformin on BPDCN cells, we investigated whether metformin modulates mitochondrial OXPHOS and glycolysis. Given the central role of mitochondrial metabolism in cancer cell survival and proliferation, we employed the Seahorse XF96 analyzer, a gold-standard tool for real-time measurement of cellular metabolic fluxes. This approach allows us to simultaneously assess oxygen consumption rate (OCR), an indicator of mitochondrial respiration, and extracellular acidification rate (ECAR), a proxy for glycolytic activity. The use of this technology was critical to capture dynamic changes in metabolic pathways and to provide mechanistic insights into metformin effects.

**Figure 6:**
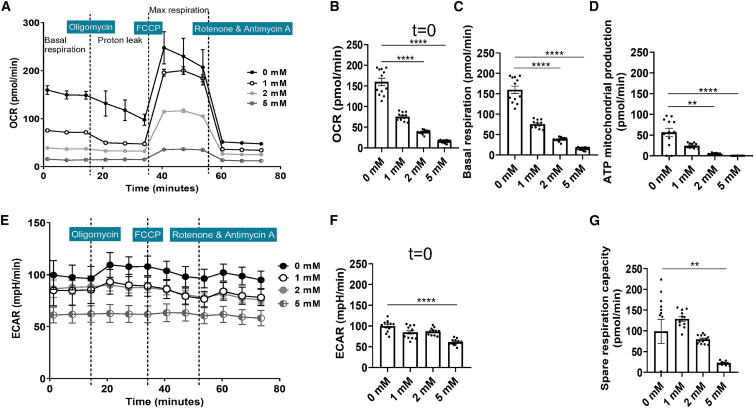
Metformin inhibits mitochondrial respiration in BPDCN cells CAL-1 cells were treated with metformin (0–5 mM) 24 h then the Cell Mito stress test kit was used to measure different parameters of mitochondrial respiration (*n* = 12 wells/group). (A) and (B) oxygen consumption rate (OCR) profile, (C) basal respiration, (D) ATP mitochondrial production, (E) and (F) the extracellular acidification rate (ECAR) and (G) spare respiration capacity (which consists in the difference between max respiration and basal respiration, see panel (A) were measured using the Seahorse XF96 Analyzer. B, C, and D correspond to t = 0 in A, and F corresponds to t = 0 in E, *i*.*e*., 24 h after metformin treatment. The sequential addition of oligomycin, FCCP, and a mix of rotenone/antimycin A, was performed at the indicated time points, as described in the Material and Methods section. (A) and (E) represent the whole kinetics of a representative experiment for OCR and ECAR, respectively. Histograms represent the mean ± SEM for 12 wells per condition. *p* value is calculated using one-way ANOVA (more than two groups). ∗∗*p* < 0.01, ∗∗∗∗*p* < 0.0001.

CAL-1 cells were treated with increasing non-cytotoxic concentrations of metformin (0–5 mM) for 24 h, followed by the mitochondrial stress assay (*n* = 2, 12 wells/condition). We observed a significant reduction in cellular oxygen consumption in CAL-1 cells treated with metformin in all states (basal respiratory, proton leak, and maximal respiratory capacity) (Figure 6A). Analysis revealed that metformin decreased oxygen consumption (Figure 6B). This diminution led to the significant reduction of basal respiration (Figure 6C). Thus, metformin inhibited mitochondrial respiration in a concentration-dependent manner. Upon injection of the ATP synthase inhibitor oligomycin the level of the ATP production decreased in a concentration-dependent manner of metformin (Figure 6D). This indicates that, in CAL-1 cells treated with metformin, mitochondria cannot provide the energy requirements. Concurrently, we observed a reduction in basal ECAR (Figures 6E and 6F) suggesting that metformin also impairs glycolytic activity. Seahorse assay showed that metformin inhibited the spare respiration capacity (Figure 6G), indicating the loss of the capacity of CAL-1 cells to respond to an energy demand. As shown in Figure S4A, metformin reduced basal OCR in GEN2.2 cells in a concentration-dependent manner, mirroring the effects observed in CAL-1 cells for basal OCR. These results suggest that metformin induced a metabolic shift in BPDCN cells, reducing their reliance on mitochondrial respiration and impairing their ability to utilize glycolysis as an alternative energy source. CAL-1 cells shifted their metabolism from an energetic profile to a more quiescent one in a concentration-dependent manner (as shown in the energy profile graph in Figure S4B). This dual inhibition of OXPHOS and glycolysis may likely contribute to the cytotoxic effects of metformin, as cancer cells often depend on these pathways for survival and proliferation.

### Metformin slightly limits BPDCN progression *in vivo*

**Figure 7:**
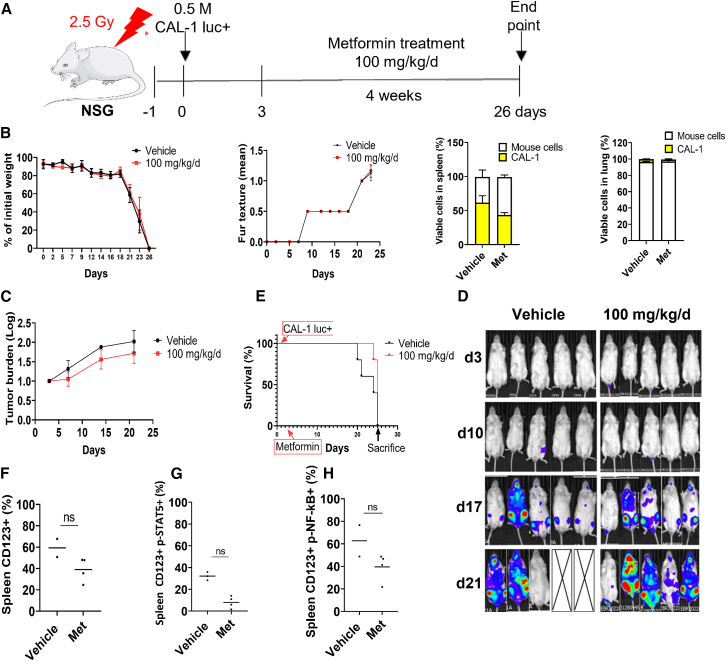
*In vivo* metformin tends to limit tumoral progression by BPDCN, to extend mouse survival as well as to limit spleen infiltration by BPDCN and to inhibit NF-κB p65, and STAT5 signaling pathways (A) A diagram describing the *in vivo* study, NSG mice were irradiated (2.5 Gy) and 18 h later, inoculated intravenously with 0.5 × 10^6^ Luciferase-expressing CAL-1 cells (LUC^+^ CAL-1). Treatment was started on day 3 (d3) after the LUC^+^CAL-1 cell inoculation with metformin (100 mg/kg/day intraperitoneally) given for 5 days/week for 4 weeks (*n* = 5 mice). Mice injected with phosphate-buffered saline (PBS) over the 4 weeks were used as the control (*n* = 5 mice). (B) The potential toxic side effects of metformin in NSG mice were assessed by monitoring changes in body weight and mouse fur texture every two days. The mice weight and mouse fur texture graphs receiving the indicted treatment are represented. The viability of mouse cells (spleen and lungs, white bars) and CAL-1 line (yellow bars) was assessed with fixable viability Dye eFluor solution using flow cytometry. CAL-1 cells were identified using human CD123 expression, while mouse cells were negative. Of note, CAL-1 cells do not infiltrate mouse lungs. (C) *In vivo* kinetics of tumor cell growth following the LUC^+^ CAL-1 cells, treated with metformin (Red) or with PBS (Black). Histogram represents the mean ± SEM for (D) luminescence of tumor-bearing mice from day 3 to day 21. (E) Kaplan Meier survival curves of mice receiving the indicted treatment. Overall survival of BPDN-inoculated mice treated with metformin (red) or with PBS (black) is shown. At the end of the *in vivo* experiments, spleens were collected from the two surviving control mice and the four surviving treated mice and were assessed for LUC^+^ CAL-1 cell quantification and phosphorylated NF-κB p65, and STAT5 expression. Cells were stained with human CD123, as well as intracellular p-NF-κB, and p-STAT5 prior to analysis by flow cytometry. The percentage of (F) CD123, (G) p-STAT5, and (H) p-NF-κB positive cells isolated from spleens of metformin-treated mice and PBS-treated mice, was quantified. Histograms represent the mean ± SEM for one experiment. ns: no significant difference.

To validate the *in vitro* findings and assess the therapeutic potential of metformin in a more physiologically relevant context, we employed a xenograft model using NSG mice. Luciferase-expressing CAL-1 cells (CAL-1 luc^+^) were injected into mice, which were then treated with metformin (100 mg/kg/day) or the same volume of PBS as a control. Metformin or vehicle was administered every day except weekends for a period of four weeks (Figure 7A).

The side effects of metformin were assessed by monitoring the total body weight and fur texture of mice in both treated and non-treated groups, every two days, revealing no differences between the groups, with no observed weight loss until day 26 or significant fur texture deterioration in both conditions. As severely immunodeficient mice, NSG mice are deficient in T, B, and NK cells. Their spleen contains randomly distributed CD45^+^ leukocytes and hematopoietic cells related to the extramedullary hematopoiesis present in mouse spleens.^22^ While no macrophage can be detected in the spleen, some NSG mice exhibit some alveolar macrophage aggregates in their^22^ lungs. The viability of mouse cells present in the lungs and the spleen was assessed by flow cytometry based on forward *vs* side scatter gating and exclusion of human CD123 expression. Metformin did not significantly affect the viability of mouse cells. These results indicated that the metformin was safe *in vivo* in the NSG mouse context (Figure 7B). While metformin treatment led to a non-significant reduction in tumor burden compared to the vehicle control group (Figures 7C and 7D), a slight (but not significant) improvement in overall survival was observed (Figure 7E). These findings suggest that metformin may have a limited impact on tumor progression and survival in this CAL-1 xenograft model.

To further investigate the antitumor effects of metformin on BPDCN, we quantified the infiltration of human CD123^+^ BPDCN cells in the spleen, as described.^23^^,^^24^ Metformin treatment reduced the percentage of human CD123^+^ cells from 59% ± 8.5% in PBS-treated mice to 38.9% ± 5.6% in metformin-treated mice (Figure 7F). However, a non-significant reduction in tumor cell infiltration in the spleen was observed (Figure 7B), probably due to the small number of surviving mice. We observed a slight cytotoxic effect on CALl-1 cells, but not on the mouse cells, suggesting that metformin has a selective effect for BPDCN cells.

To confirm our *in vitro* previous data, we evaluated the activation of selected molecules in spleen-infiltrating CD123^+^ CAL-1 cells (Figure 7F). Metformin treatment significantly reduced the phosphorylation levels of NF-κB p65 and STAT5 from 62% ± 13.9% to 39% ± 6%, and from 32% ± 3.8% to 8% ± 2%, respectively, compared to the PBS control group (Figures 7G and 7H). However, again, no significant reduction was observed for NF-κB p65 and STAT5 phosphorylation. These results align with our *in vitro* findings and suggest that metformin may modulate critical survival pathways in BPDCN cells *in vivo*.

## Discussion

BPDCN is a hematodermic neoplasia with poor prognosis.^25^^,^^26^ Its treatment represents a main challenge.^27^ Metformin remains the largely used anti-diabetic treatment in the world that has been reported to reduce the risk of cancer in patients with diabetes.^5^^,^^28^^,^^29^ However, multiple observational studies have demonstrated that metformin has a similar protective effect of non-diabetic patients with cancer.^7^^,^^30^^,^^31^ In hematological neoplasms, antineoplastic effects of metformin have been described in acute myeloid leukemia (AML)^32^^,^^33^^,^^34^ and in acute lymphoblastic leukemia.^6^ In our study, we tried to determine the impact of metformin on different BPDCN cells *in vitro*. CAL-1 cells were mainly used, and data were confirmed in GEN2.2 cells (*i*.*e*., effect on cell death, proliferation and on AMPK, Akt, mTOR, and NF-κB p65 phosphorylation) and in three primary BPDCN cells (*i*.*e*., effect on cell death, on Akt, mTOR, NF-κB p65, and STAT3 signaling, Table S1). We confirmed that CAL-1, GEN2.2 (Figure 1), and PDX cells (Figure S1A) were sensitive to metformin. IC50 in BPDCN cells after 24 h is in accordance with the results previously obtained in other leukemic cells.^35^^,^^36^ This drug decreased cell proliferation and viability in a concentration-dependent manner. These results are coherent with previous studies, which demonstrated that metformin induced inhibitory effects on cell viability and induced apoptosis in various malignancies such as breast,^37^ colorectal cancer,^38^ and leukemia.^2^^,^^5^^,^^39^ A trend was also observed *in vivo*, where metformin treatment led to a slight reduction in BPDCN cells in immunodeficient mice, although the difference was not statistically significant (Figures 7B and 7F). The concentrations used *in vitro* (1–10 mM) exceed plasma levels in diabetic patients (10–40 μM). However, these doses are standard in preclinical cancer models to compensate for low expression of organic cation transporters (OCTs) in cultured cells and to mimic the mitochondrial accumulation of metformin that occurs *in vivo*. *In vivo*, metformin accumulates in different tissues such as the liver and bone marrow at concentrations exceeding 100 μM, and its mitochondrial concentration is estimated to reach the millimolar range. Therefore, the *in vitro* doses employed in our study are appropriate to reproduce the intracellular bioenergetic effects of metformin relevant to tumor cells.^40^^,^^41^

Further exploring the cell death mechanism using CAL-1 cells, we showed that metformin induces apoptosis in a concentration-dependent manner and confirmed using western blot and live-cell imaging that metformin triggers caspase-3, caspase-7, and caspase-9 activity (Figure 2). Consistent with these results, previous studies report that metformin significantly induces apoptosis in AML.^5^^,^^42^ Moreover, metformin triggers the intrinsic apoptotic pathway in CAL-1 cells, as attested by caspase-9 cleavage. Engagement of this pathway was previously reported with a human gastric adenocarcinoma cell line.^43^ Metformin did not affect normal CD34^+^ hematopoietic stem cells,^15^ supporting its use in patients.

In several studies using different cancer cells, AMPK triggered by metformin was described as an important regulator of cellular energy homeostasis^28^^,^^44^^,^^45^ and to inhibit anabolic processes essential for cancer cell growth.^3^^,^^41^^,^^44^ Our results demonstrated that the AMPK pathway is activated by metformin in the CAL-1 (Figures 3A and 3B) and in GEN2.2 (Figures S2A and S2B) BPDCN cell lines. Both AMPK dependent^6^^,^^44^^,^^46^ or AMPK-independent^47^^,^^48^ mechanisms have previously been described in hematological malignancies.^3^^,^^35^^,^^49^ To determine if AMPK is involved in metformin-induced apoptosis in BPDCN, we blocked AMPK activity using the CC inhibitor. Our results demonstrated that AMPK inhibition did not significantly reverse the cytotoxic effects of metformin in CAL-1 cells (Figures 3C and 3D). Consequently, the mechanism of metformin-induced cell death is mainly independent of AMPK activation. Similar results were observed in various cancers, including leukemia.^6^^,^^32^^,^^42^

A common biochemical feature among acute leukemia is the abnormal and constitutive activation of the Akt/mTOR pathway.^50^^,^^51^ As suggested by previous studies, targeting the Akt/mTOR pathway may exert apoptotic and antiproliferative effects in hematological malignancies.^12^^,^^32^^,^^50^ We showed that metformin significantly decreased p-Akt and p-mTOR expression levels in BPDCN cell lines (CAL-1, GEN2.2) and BPDCN PDX (Figure 4, see also Table S1). This indicates that metformin-mediated alterations of the Akt/mTOR signaling pathway could be associated with apoptosis induction and/or reduced cell proliferation in BPDCN.

Involvement of constitutive NF-κB p65 activation in BPDCN cell lines (CAL-1 and GEN2.2 cells) and primary BPDCN cells has been previously reported.^11^ Here, metformin inhibited NF-κB p65 phosphorylation in all BPDCN cells lines and one BPDCN PDX. Constitutive expression of STAT3^13^ and STAT5^52^^,^^53^ was also reported to participate in leukemic cell survival and proliferation. In our study, metformin was able to downregulate p-STAT3 and p-STAT5 expression levels in CAL-1 and p-STAT3 in one primary BPDCN (Table S1). This inhibitory effect of metformin on STAT3 and STAT5 phosphorylation was reported in several cancers, thus breast cancer^13^ and leukemia.^5^^,^^7^^,^^50^ Thus, metformin affects several signaling pathways in BPDCN.

Metabolic reprogramming from OXPHOS to glycolysis is a hallmark of cancer,^54^ a phenomenon referred to as the Warburg effect.^55^ As suggested, treatment of AML with metformin may also take advantage of metabolic difference between tumoral and normal tissues.^46^ Metformin has been shown to inhibit OCR in colon cancer cells, which is consistent with the inhibition of OXPHOS.^56^ These authors propose that a decrease in OXPHOS corresponds to depletion of ATP supply and could force the cells to engage survival and increased glycolysis leading eventually to cell death.^56^ We used a quantitative real-time measurement of OXPHOS and glycolysis in CAL-1 cells and showed that metformin decreased OCR, ECAR, mitochondrial ATP production, and spare respiratory capacity. Similar inhibition of basal OCR was observed for GEN2.2 cells. However, we were not able to assess the effects of metformin in the other metabolic parameters, since GEN2.2 cells exhibit a more quiescent metabolic profile than CAL-1 cells. Nevertheless, this may suggest that inhibition of mitochondrial activity by metformin could contribute to decreased cell viability and/or proliferation observed in BPDCN cells. This inhibition of mitochondrial function aligns with the role of metformin as a metabolic modulator and underscores its ability to impair energy production in leukemic cells, thereby compromising their survival and proliferative capacity.^2^^,^^3^^,^^5^^,^^57^

Several *in vivo* studies showed the protective effect of metformin against different hematological malignancies, such as AML^5^^,^^46^ and lymphoma^58^ but never in BPDCN. Here, metformin produced a slight non-significant antitumor effect in a xenograft model using CAL-1 cells. In survivor mice, we observed an inhibition of phosphorylated NF-κB p65 and STAT5 induced by metformin *in vivo* in CAL-1 cells. Our results demonstrate a cytotoxic effect on the CAL-1 line but not on the mouse cells present in the spleen and the lungs. However, effects of metformin on CAL-1 cells did not achieve statistically significant levels due to the limited number of survivor mice (2 in the PBS-treated group *vs*. 4 in the metformin-treated group). Different preclinical (*in vivo*) studies have shown that metformin is a potential therapeutic approach in hematological malignancies, particularly through its impact on key pathways such as Akt/Mtor.^5^^,^^59^^,^^60^ Our findings suggest that metformin may inhibit key survival signaling pathways in BPDCN. These effects—although non-significant—are consistent with our *in vitro* observations, further supporting the capacity of metformin to modulate BPDCN progression. However, its impact on overall tumor progression and survival is limited in the BPDCN xenograft model. This attenuated modulation of metformin’s antitumor effect could be explained by the macrophage deficiency in NSG mice. Indeed, the antitumor activity of metformin has been linked to its ability to induce macrophage polarization toward a pro-inflammatory M1 phenotype, a mechanism that is absent or strongly reduced in this immunodeficient model.^61^ Additional *in vivo* studies using other models and an in-depth analysis of the effects on the normal counterpart of BPDCN (*i*.*e*., pDCs) are needed. In humans, pDCs represent less than 0.5% of circulating blood cells, making their isolation from peripheral blood complex. Furthermore, once isolated, these cells die rapidly except if their survival factor IL-3 is added. This renders difficult the analysis of metformin on pDCs. In contrast, the CAL-1 xenograft is a well-established and widely used model for BPDCN,^11^^,^^18^ not only by our group.^62^^,^^63^

### Limitations of the study and future perspectives

Our findings suggest the therapeutic potential of metformin in targeting BPDCN. Through combined *in vitro* and *in vivo* analyses, we demonstrated that metformin exerts potent anti-proliferative and cytotoxic effects on BPDCN cells *in vitro*. These effects could be mediated by a multifaceted mechanism involving AMPK activation, inhibition of the Akt/mTOR and NF-κB p65 pathways, downregulation of STAT3 and STAT5 signaling, and a profound metabolic reprogramming characterized by dual inhibition of oxidative phosphorylation and glycolysis. However, the impact of metformin on overall tumor progression and survival in the BPDCN xenograft model remained limited.

This study provides preliminary, proof-of-concept evidence for the metabolic vulnerability of BPDCN but also highlights important biological and technical sources of variability. BPDCN represents a heterogeneous disease with malignant cells that differ at molecular and phenotypic levels.^10^^,^^11^ Differences in metformin sensitivity across the models (*i*.*e*., BPDCN cell lines, PDX) likely reflect both intrinsic and experimental factors. While CAL-1 cells represent a stable and reproducible BPDCN model, its limitation is that it is an established cell line. Concerning GEN2.2 cell line, it requires to grow on MS-5 feeder cells.^64^ GEN2.2 cells were plated on poly-L-lysine-coated plates without stromal support in Seahorse assays, which may have affected their metabolic behavior and may explain their quiescent metabolic profile. PDX cells display distinct metabolic and signaling profiles that can influence treatment responses. Likewise, PDX cells, which are particularly fragile even if they have the advantage to represent primary cells and to cover the heterogeneity of BPDCN, were treated for short durations to minimize stress-induced cell death-likely contributing to the absence of statistically significant changes in AKT/mTOR and STAT5 signaling. Altogether, molecular and phenotypic heterogeneity of the BPDCN cells used in this study may explain differences in metformin sensitivity (please refer to Table S2).

Given the intrinsic heterogeneity of BPDCN, testing therapeutic strategies across multiple models—including GEN2.2, additional PDXs, and primary patient cells—will be crucial to fully capture disease diversity. Moreover, improving the *in vivo* system through the use of humanized mouse models would enable evaluation of the contribution of immune components, such as M1 macrophage polarization, to metformin responses. In conclusion, the variability in metformin responses across models does not diminish the validity of our findings but rather highlights the importance of testing therapies across multiple systems to capture the full spectrum of BPDCN biology. Future studies will aim to stratify BPDCN subtypes based on pathway activation profiles to identify patients most likely to benefit from metformin-based therapies.

We acknowledge that using a humanized mouse model with a reconstituted human immune system would be ideal to assess the full therapeutic potential of metformin in BPDCN. However, such models are technically demanding, costly, and are not available in our laboratory. Our current approach should therefore be viewed as a proof-of-concept demonstrating that metformin can directly target BPDCN cells *in vivo*, albeit with limited efficacy in the absence of immune engagement. Future studies are required using humanized mice (e.g., NSG-SGM3 mice engrafted with human hematopoietic stem cells) to evaluate the contribution of macrophage polarization and other immune mechanisms to metformin’s antitumor effects.

Azacitidine, a DNA hypomethylating agent, may sensitize cells to metabolic stress,^65^ and metformin could enhance its pro-apoptotic effects through epigenetic-metabolic interaction.^66^ Metformin may also enhance the activity of venetoclax by downregulating anti-apoptotic proteins such as Mcl-1 and BCL-xL,^42^^,^^67^ which is particularly relevant in BPDCN, where BCL-xL upregulation contributes to venetoclax resistance.^68^ in addition metformin induced metabolic reprogramming might increase the susceptibility of BPDCN cells to tagraxofusp, a CD123-targeted cytotoxin. Although these synergetic mechanisms have been suggested in other hematologic malignancies,^42^^,^^69^ they still require validation in BPDCN models. Further combination studies are needed to optimize dosing, scheduling, and safety. Given its favorable safety profile, metformin represents a promising adjuvant to existing therapies and may help reduce the required doses of more toxic agents.

## Resource availability

### Lead contact

Requests for further information and resources should be directed to and will be fulfilled by the lead contact, Mourad Aribi, (mourad.aribi@univ-tlemcen.dz).

### Materials availability

This study did not generate new unique reagents.

### Data and code availability

• All data reported in this paper will be shared by the lead contact upon request.
• This paper does not report original code.
• Any additional information required to reanalyze the data reported in this paper is available from the lead contact upon request.

## Acknowledgments

The authors acknowledge the UMR RIGHT. We also thank DImaCell Imaging Platform (Université Marie et Louis Pasteur, UMR RIGHT, 25000 Besançon, France) for technical support during IncuCyte S3 live-cell analysis and confocal microscopy experimentations. This work is supported by the Agence Nationale de la Recherche (ANR) under the program “Investissements d’Avenir” (ANR-11-LABX-570 0021-LipSTIC), the Etablissement Français du Sang Bourgogne Franche-Comté, UMR RIGHT, SAIC, University Marie et Louis Pasteur (France), Laboratory of Applied Molecular Biology and Immunology, W0414100, and University of Tlemcen (Algeria).

## Author contributions

M.A. and P.S. conceptualized the project, provided overall guidance, jointly supervised all specific aspects of the project, including experimental design and the development of protocols, data analysis, and the overall integration of research findings across different stages of the study, reviewed the results and graphical illustrations, and edited the final version of the manuscript. S.P. provided essential project support, ensuring the smooth progression of the experiments. Z.M. conducted the experiments, including *in vitro* and *in vivo* assays, designed and executed the experimental protocols, analyzed the data, and drafted the original version of the manuscript, integrating key findings and contributing to the formulation of the study’s conclusions. L.D.Z., M.F., M.P., and M.V. performed *in vivo* experiments. F.G.-O., M.F., and S.B. contributed to the establishment, characterization, and maintenance of PDX models. G.R. optimized staining conditions and provided fluorescent antibodies for real-time imaging using the IncuCyte live-cell analyzer. S.B. played a key role in designing and optimizing flow cytometry experiments, providing valuable suggestions for experimental protocols and data interpretation. V.M. provided key reagents and expertise for the optimization of confocal microscopy experiments and imaging. M.A. and P.S. share the last author position (co-last authors). S.P., F.G.-O., G.R., M.V., and M.F. commented on the manuscript. P.M.-L. and Y.G. help to perform experiments after the revision process.

## Declaration of interests

The authors declare no competing interests.

## STAR★Methods

### Key resources table

**Table:** 

REAGENT or RESOURCE	SOURCE	IDENTIFIER
**Antibodies**		

7AAD	Beckman Coulter	Cat#A07704. Cat# 300002
Actin	Sigma	Cat#A5441; RRID: AB_476744
AMPKα	Cell Signaling	Cat#2532 S; RRID: AB_330331
Annexin V FITC Apoptosis Detection Kit I	BD Biosciences	Cat#556547; RRID: AB_2869082
Annexin-V	Sony	Cat#3804530
Caspase 3	Cell Signaling	Cat#9662 S; RRID: AB_331439
Caspase 9	Cell Signaling	Cat#9502 S; RRID: AB_2068621
CD123	BD Biosciences	Cat#340545; RRID: AB_400052
CD123	Sony	Cat#2130050
CD303	BD Biosciences,	Cat#748000; RRID: AB_2872461
Cleaved caspase 3	Cell Signaling	Cat#9661 S; RRID: AB_2341188
Cleaved caspase 9	Cell Signaling	Cat#9501 S; RRID: AB_331424
Donkey Anti-Rabbit IgG (H + L), (Alexa Fluor 488 Conjugate)	Invitrogen	Cat#A32790; RRID: AB_2762833
fixable viability Dye eFluor	Invitrogen	Cat#65-0863-14
Goat anti rabbit HRP	Cell Signaling	Cat#7074 P2; RRID: AB_2099233
Incucyte® Caspase-3/7 Green Dye	Startious	Cat#4440
Incucyte® Cytotox Red Dye	Startious	Cat#4632
*p*-Akt	Invitrogen	Cat#44-621G; RRID: AB_2533699
*p*-AMPKα	Cell Signaling	Cat#2535 S; RRID: AB_331250
*p*-mTOR	Invitrogen	Cat#44-1125G; RRID: AB_2533580
p-NF-kB	Invitrogen	Cat#MAS-15160; RRID: AB_10983078
p-STAT3	Cell Signaling	Cat#9145 S; RRID: AB_2491009
p-STAT5	Cell Signaling	Cat#9314 S; RRID: AB_2302702
Sheep anti mouse HRP	Jackson	Cat#515-035-062; RRID: AB_2340296

**Chemicals, peptides, and recombinant proteins**		

Compound C	Merck KGaA	Cat#171260-1 MG
Human IL-3	Miltenyi	Cat#130-096-071
Luciferin	Promega	Cat#P1043
Metformin (*in vitro*)	Merck KGaA	Cat#317240-5 GM
Metformin (*in vivo*)	Medchem express (MCE)	Cat#HY-17471 A
poly-D-lysine	Merck KGaA	Cat#P7886-10 MG
PP242	Merck KGaA	Cat#475988-5 MG
R848	Invivogen	Cat#tlrl-r848

**Critical commercial assays**		

Cell Mitochondrial Stress Test assay	Agilent	Cat#103015-100
Glycolytic Rate Assay	Agilent	Cat#103344-100

**Experimental models: Cell lines**		

CAL-1	Dr. Maeda, Nagasaki University, Japan	RRID:CVCL_5G46
GEN2.2	Dr Laurence Chaperot, EFS, France	Patent #0215927, RRID:CVCL_5G44
MS-5	Maxime Fredon, Prof. Francine Garnache-Ottou, EFS Besançon, France	RRID:CVCL_2128
NGS mice	The Jackson Laboratory	N/A
PBMC	EFS Besançon, France	N/A
PDX	Sabeha Biichle, Prof. Francine Garnache-Ottou, EFS Besançon, France	Primary BPDCN cells

**Software and algorithms**		

FACSDiva	BD Biosciences	N/A
GraphPad 9.0 Prism	GraphPad	N/A
ImageJ	public domain software	N/A
Kaluza	Beckman	N/A
Zen 3.3 Blue	Carl Zeiss	N/A

### Experimental model and study participant details

All animal experiments were conducted in compliance with institutional guidelines and approved by the Veterinary Services for Animal Health & Protection, Ministry of Agriculture, Paris, France (Protocol #2021-004-0 A-12 PR). Every effort was made to minimize animal suffering, discomfort, and stress throughout the study. Animals were housed under controlled environmental conditions (temperature, humidity, and light/dark cycle). Health and welfare were closely monitored daily by trained personnel to ensure humane treatment and early detection of any signs of illness.

### Method details

#### Cell lines, culture conditions, and reagents

The human BPDCN cell lines, CAL-1 (provided by Dr. Maeda, Nagasaki University, Japan)^70^ and GEN2.2 (patent #0215927, EFS, France)^64^ were used throughout the study. Some data were confirmed in three different BPDCN PDX cells (P193B-M1, P224B-M2 and P178M-M1, consisting of primary BPDCN cells [sample collection authorization numbers: #DC- 2008–713 and #DC 2016–2791, approval of the local ethics committee: CPP Est II 07/02/2017, Besançon, France] from three different patients with BPDCN that were used for amplification of primary cells in immunodeficient NSG mice to produce BPDCN PDX.^18^ These cells have been fully characterized according to current recommendations for the diagnosis of BPDCN).^71^^,^^72^ Based on the analyzed phenotypic markers and next-generation sequencing (NGS) analysis of the two lines and the three PDXs, differences were observed attesting for molecular and phenotypic heterogeneities (Table S2). According to the initial definition of BPDCN (CD4^+^, HLA DR^+^, CD123^+^, CD45RA^+^, CD11c^‒^ and CD13^‒^)^64^ and the two successive updates with the diagnosis strategy,^71^ scoring first the CD4^+^ CD56^+/−^ MPO^‒^ cCD3^‒^ cCD79a^‒^ CD11c^‒^ profile and then the CD123^high^, BDCA-2 and BDCA-4 expression, all BPDCN cells exhibited some particularities (CAL-1 cells CD11c, CD38 and CD33 expression; GEN2.2 cells CD7 expression but not CD56, P178M-M1 cells CD33 expression, P193B-M1 CD117, CD36, CD141 and cTdt expression, and P224B-M1 CD2 expression). TET2 mutations, which are found in BPDCN^73^ were found in P178M-M1 cells and GEN2.2 cell lines (Table S2). All cells were maintained at 37°C under 5% CO2. CAL-1 and BPDCN PDX were cultured in complete RPMI 1640 Glutamax medium (Gibco, Paisley, United Kingdom) supplemented with 10% heat-inactivated fetal calf serum (FCS) (Gibco) and 1% Penicillin-Streptomycin (10 000 U/mL, Gibco).^11^^,^^70^ GEN2.2 cells were grown on irradiated MS-5 (DSMZ, #ACC 441) feeder cells in RPMI 1640 Glutamax medium supplemented with 1% sodium pyruvate (Gibco), 1% non-essential amino acids (Gibco), 10% FCS, and 1% Penicillin-Streptomycin.^11^^,^^64^ To evaluate the potential cytotoxic effect of metformin on non-transformed healthy cells, we evaluated cell viability in 3 different human peripheral blood mononuclear cells (PBMCs) following metformin exposure, as they widely used in translational immunology and functional assays to model the human immune response,^74^^,^^75^ and provide a physiologically relevant model for studying drug responses in primary immune populations.^76^ This analysis complements the dose-response results obtained in CAL-1 and GEN2.2 cell lines. PBMC were collected from healthy donors at the Etablissement Français du Sang (EFS, Besançon, France) as apheresis kit preparations after informed consent and according to the collection agreement AC-2020-4129. Cells were cultured in RPMI-1640 medium (Fisher Scientific, 11544526) with 10% human serum (local production).^77^ Metformin for *in vitro* assay (Sigma-Aldrich, Saint-Quentin Fallavier, France) was prepared as a 100 mM stock solution in phosphate-buffered saline (PBS). All cell lines were checked monthly for mycoplasma contamination. Metformin hydrochloride for *in vivo* assay (Medchem express (MCE), Stockholm Sweden). The AMPK inhibitor Compound C (CC) and the mTORC1/C2 inhibitor (PP24) were obtained from Merck KGaA (Darmstadt, Germany). R848 (invivogen, Toulouse, France) was dissolved in water endotoxin-free. Human IL-3 was from (Milteneyi Biotec, Bergisch Gladbach, Germany).

#### Flow cytometry assay

Cell death was assessed using flow cytometry on a FACS Canto II instrument (BD Biosciences, Le Pont de Claix, France). Cells were stained with FITC-conjugated Annexin-V (BD Biosciences) and 7-aminoactinomycin D (7-AAD) (Beckman Coulter, Roissy, France) according to the manufacturer’s protocols. CAL-1 and GEN2.2 cell lines were seeded at a density of 5 × 10^5^ cells/mL and treated with increasing concentrations of metformin (0–50 mM) for 24 h at 37°C under 5% CO_2_, either alone or in combination with 1 μM of the AMPK inhibitor Compound C (CC) for 2 h pretreatment.^49^ Data acquisition and analysis were performed using the DIVA 6.2 software (BD Biosciences). Cell viability for PBMC from healthy donors was assessed using Annexin-V– (Sony, San Jose, California, USA)/7-AAD- (Beckman Coulter, Roissy, France) according to the manufacturer’s instructions and analyzed using flow cytometry Beckman Coulter CytoFLEX LX flow Cytometer and analyzed through Kaluza software (version 2.1).

Dose-response curves were fitted using nonlinear regression with a four-parameter logistic (4 PL) model

$$Y=Bottom+\frac{Top-Bottom}{1+{\left(\frac{X}{{IC}_{50}}\right)}^{HillSlope}}$$

where Y represents the percentage of viable cells, X represents metformin concentration, Top and Bottom represent the asymptotic maximum and minimum of the curve, HillSlope describes curve steepness, and IC50 is the inhibitory concentration at which viability is reduced by 50%. IC50 values were calculated using nonlinear least-squares regression in GraphPad Prism (version 9.0; GraphPad Software, Inc., San Diego, CA, USA) software following standard pharmacological curve-fitting procedures^46^^,^^78^^,^^79^ Goodness-of-fit was assessed using the coefficient of determination (R^2^) and residual distribution. Data are presented as mean ± SEM of independent biological triplicates.

#### Western blot assay

To investigate the expression of AMPK and Caspase-3/9 (to confirm apoptosis observed by flow cytometry and IncuCyte live-cell analysis), Western blotting was performed. CAL-1 cells (7 × 10^6^) were treated with increasing concentrations of metformin (0–50 mM) for 6 h.^80^ To confirm the AMPK-dependent effects of metformin, CAL-1 cells were pre-treated with 1 μM of the AMPK inhibitor Compound C (CC) for 2 h, followed by incubation with 10 mM of metformin for 24 h. CAL-1 and Gen2.2 cells were pre-treated with 1 μM of the mTORC1/C2 inhibitor (PP24, Merck KGaA, Darmstadt, Germany) for 2 h, followed by incubation with 10 mM of metformin for 24 h. Cells were lysed in a sample buffer containing 2% sodium dodecyl sulfate (SDS) in 125 mM Tris-HCl (pH 6.8). Protein concentrations in the supernatants were quantified using the Pierce Bicinchoninic Acid (BCA) Protein Assay kit (Bio-Rad, Santa Rosa, CA) according to the manufacturer’s instructions. Equal amounts of protein were separated on 8.5% or 12% SDS-PAGE gels and transferred to polyvinylidene difluoride (PVDF) membranes (GE HealthCare, Strasbourg, France). Membranes were blocked with 6% non-fat milk for 1.5 h at 25°C and then incubated overnight at 4°C with primary antibodies against AMPK, phospho-AMPK, Caspase-3, cleaved Caspase-3, Caspase-9, and cleaved Caspase-9 (key resources table) (Cell Signaling Technology, Danvers, MA). Mouse anti-human β-actin (Sigma-Aldrich) was used as a loading control. After washing three times with Tris-buffered saline containing 0.1% Tween 20 (TBST), membranes were incubated for 1.5 h at 25°C with horseradish peroxidase (HRP)-conjugated goat anti-rabbit or sheep anti-mouse secondary antibodies (Cell Signaling Technology). Protein bands were visualized using the Clarity Western ECL Substrate (Bio-Rad) on a bioluminescence imager (Vilber-Lourmat, Marne-la-Vallée, France) and analyzed using BIO-1D Advanced software. The quantification was performed using ImageJ software.

#### Immunofluorescence and confocal imaging

To investigate the inhibitory effects of metformin on the expression of phosphorylated proteins RelA (p-NF-κB p65), *p*-mTOR, *p*-Akt, p-STAT3, and p-STAT5 in CAL-1, GEN2.2 and three different PDX cells, confocal microscopy analysis was performed. For *p*-mTOR and *p*-Akt expression, CAL-1 and GEN2.2 cells were treated with or without 5 mM metformin for 6 h. To assess nucleus p-NF-κB p65 expression, CAL-1 and GEN2.2 cells were incubated with or without 5 mM metformin for 24 h, in CAL-1 followed by TLR7 stimulation using R848 (1 μg/mL) for 6 h. For p-STAT3 and p-STAT5 evaluation, CAL-1 cells were treated with metformin (0–5 mM) for 24 h, and then stimulated with 10 ng/mL IL-3 for 30 min.^81^ BPDCN PDX cells were incubated with or without 5 mM metformin for 6 h without TLR7 stimulation. At the end of the treatments, cells were washed with PBS and immobilized on glass microscope slides using Cytospin centrifugation. Cells were fixed and permeabilized in a methanol bath at −20°C for 20 min. After washing, cells were incubated overnight at 4°C with primary monoclonal antibodies against *p*-mTOR, *p*-Akt and p-NF-κB p65 (Invitrogen, Rockford, IL), p-STAT3 and p-STAT5 (Cell Signaling Technology) (key resources table). Following three washes with PBS containing 0.5% Bovine Serum Albumin (BSA), cells were incubated for 1 h at room temperature with Alexa Fluor 488-conjugated Donkey anti-Rabbit IgG (H + L) secondary antibody (key resources table) (Invitrogen). After additional washes, slides were mounted using mounting media containing 4′,6-diamidino-2-phenylindole (DAPI) (Sigma-Aldrich) or Hoechst (Invitrogen). This protocol is adapted from the work of Ceroi.^80^ Fluorescent images were acquired using an LSM800 confocal microscope (Carl Zeiss, Oberkochen, Germany), and fluorescence quantification was performed using Zen 3.3 Blue software (Carl Zeiss).

#### Real-time live-cell imaging

To monitor cytotoxicity and apoptosis in real-time over a 48-h period, fluorescent markers were used in conjunction with metformin treatment. Cells were maintained in their culture medium on scanning plates placed in a humidified incubator at 37°C under 5% CO_2_. For proliferation and apoptosis assays, CAL-1 cells (7 000 cells/well) were seeded into 96-well plates and treated with increasing concentrations of metformin (0–50 mM, *n* = 8 wells per concentration). After a 30-min equilibration period at room temperature, plates were transferred to the IncuCyte™ S3 Kinetic Live Cell Imaging System (Sartorius®, Göttingen, Germany) and incubated at 37°C under 5% CO_2_. Cells were imaged at 10 X magnification every 2 h for up to 48 h. For apoptosis detection, 1X IncuCyte® Caspase-3/7 Green Reagent (key resources table) (Sartorius®) was added simultaneously with metformin. For cell death assessment, IncuCyte® Cytotox Red dye (key resources table) (Sartorius®), which detects cell membrane integrity disruption was added to the treatment medium. Real-time phase-contrast, red, and green fluorescent images were captured and analyzed using IncuCyte® image analysis software (Sartorius®).

#### Seahorse technology-based real-time analysis of glycolysis and mitochondrial OXPHOS

The assay was performed to evaluate OXPHOS through the measurement of mitochondrial ATP production and oxygen consumption, as well as to determine whether cells underwent a metabolic shift to glycolysis. Real-time measurements were conducted over an 80-min period in a controlled 37°C environment without CO_2_. CAL-1 cells were treated with metformin (0–5 mM) for 24 h (12 wells per condition). Following treatment, the cells were plated in each well of a Seahorse XF96 cell culture microplate (Agilent Technologies, Centerville Road, Wilmington) pre-coated with poly-D-lysine (Sigma), according to the manufacturer’s instructions. The culture media were replaced with Agilent Seahorse XF Media supplemented with glucose, pyruvate, and L-glutamine. The plates were then equilibrated in a 37°C incubator without CO_2_ for 1 h prior to the Seahorse Cell Mitochondrial Stress Test assay (Agilent Technologies) for CAL-1 cells and submitted to OCR and extracellular acidification rate (ECAR) evaluation. Modulators of mitochondrial function were then sequentially added at the indicated time points as follows: 1.5 μM oligomycin (an oxidative phosphorylation inhibitor), 0.5 μM carbonyl cyanide-4-(trifluoromethoxy) phenylhydrazone (FCCP, a potent mitochondrial uncoupling agent), and 0.5 μM a combination of rotenone and antimycin A (an inhibitor of mitochondrial complex I and an inhibitor of mitochondrial complex III, respectively) in order to determine maximum respiration. Oligomycin, FCCP, rotenone and antimycin A were provided in the Seahorse Cell Mitochondrial Stress Test assay. Spare respiratory capacity was then defined as the difference between maximal respiration and basal respiration. Basal respiration and ATP mitochondrial production were measured 24 h after treatment with metformin. For GEN2.2 cells, the Seahorse Glycolytic Rate Assay (Agilent Technologies) was used and submitted to OCR. Modulators of mitochondrial function were then sequentially added at the indicated time points as follows: Rotenone and Antimycin A (Rot/AA) and 2-deoxy-D-glucose (2-DG). Measurements were performed using an Agilent Seahorse XFe96 metabolic flux analyzer (Agilent Technologies). Data were analyzed by Seahorse XF-96 Wave software according to manufacturer recommendations and expressed as pmol/min for OCR and mitochondrial ATP production and mpH/min for ECAR.

#### CAL-1 *in vivo* xenograft model

To determine whether the cytotoxic and inhibitory effects of metformin on NF-κB and STAT3/5 observed *in vitro* could be replicated *in vivo* using a CAL-1 cell xenograft model in immunodeficient mice. For the assay, NOD-SCID IL2Rγc-deficient (NSG) female mice (The Jackson Laboratory, Sacramento, CA), bred in our specific pathogen-free rodent facility (Agreement #D25-056-7), aged 2 to 5 months, were irradiated with 2.5 Gy and inoculated intravenously 18 h later with 0.5 × 10^6^ luciferase-expressing CAL-1 cells (CAL-1 luc+). Three days post-inoculation, mice (*n* = 5 per group^5^^,^^7^) were treated intravenously with metformin (Sigma-Aldrich) at a dose of 100 mg/kg/day or with PBS (vehicle) for 5 days per week over 4 weeks. Body weight and fur texture were assessed individually every 2 days. Fur texture was scored as follows: normal (score = 0), mild to moderate ruffling (score = 0.5), severe ruffling/poor grooming (score = 1), according to Cooke et al.^81^

Tumor progression was monitored weekly by bioluminescence imaging (IVIS Lumina Series III; PerkinElmer, Waltham, MA) following intraperitoneal injection of luciferin (VivoGlo Luciferin, #P1043, Promega, Fitchburg, WI). Survival was tracked daily, and at the endpoint, mice were sacrificed, and spleen infiltration by CAL-1 luc+ cells was quantified by flow cytometry using anti-human CD123 antibodies (key resources table; BD Biosciences). The viability of mouse cells (spleen and lungs) and CAL-1 line was assessed with fixable viability Dye eFluor solution according to manufacturer’s protocol (Invitrogen, Carlsbad, CA, USA). Protein expression levels of p-NF-κB, and p-STAT5 were analyzed using specific monoclonal antibodies (key resources table; Invitrogen).

All animal experiments were conducted in compliance with institutional guidelines and approved by the Veterinary Services for Animal Health & Protection, Ministry of Agriculture, Paris, France (Protocol #2021-004-0 A-12 PR).

### Quantification and statistical analysis

Statistical analyses were performed using GraphPad Prism (version 9.0; GraphPad Software, Inc., San Diego, CA). Data are presented as the mean ± SEM of at least three independent experiments. For comparisons involving three or more groups, a Kruskal-Wallis test (non-parametric one-way analysis-of-variance [ANOVA]) was used, while the Mann-Whitney *U* test was applied for comparisons between two groups, as the data were not normally distributed. A *p*-value of <0.05 was considered statistically significant.

## Supplemental information

Document S1. Figures S1–S4, Data S1, and Tables S1 and S2

Video S1. Stages of proliferation, apoptosis and cytotoxicity, related to Figure 1 and 2
