# Supplementary material for: Metformin dual-targets metabolism and survival pathways in BPDCN
Source: iScience. 2026 Jun 13;29(7):116323. doi: 10.1016/j.isci.2026.116323 (PMC13279768; doi:10.1016/j.isci.2026.116323)
Supplement: Document S1. Figures S1–S4, Data S1, and Tables S1 and S2 [file mmc1.pdf]

iScience, Volume *29*

Supplemental information

Metformin dual-targets metabolism

and survival pathways in BPDCN

Zineb Mekkaoui, Ludivine Dal Zuffo, Mathieu Vetter, Maxime Fredon, Margaux Poussard, Sabeha Biichle, Virginie Mougey, Patricia Mercier-Letondal, Gwenaël Rolin, Yann Godet, Sylvain Perruche, Francine Garnache-Ottou, Philippe Saas, and Mourad Aribi

Figure S1.

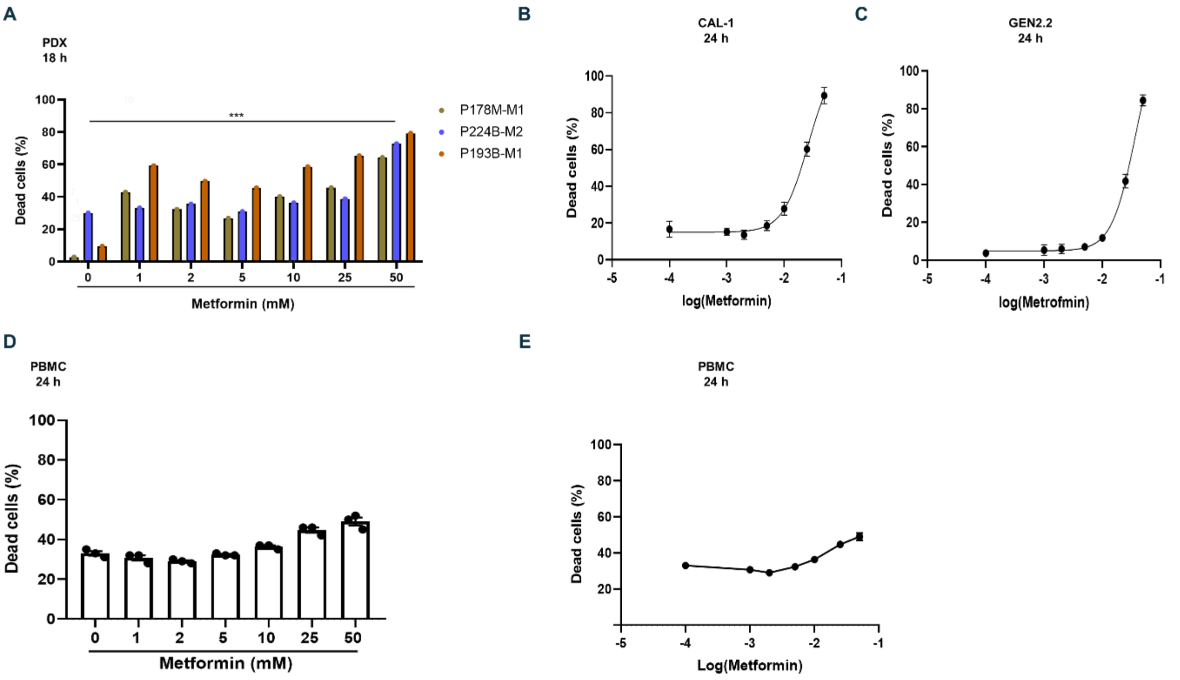

Figure S1. Metformin inhibits cell survival, proliferation in blastic plasmacytoid dendritic cell neoplasm but not in healthy cells. Three BPDCN cells (three different Patient Derived Xenografts (PDX**),** CAL-1 and GEN2.2 cells line), PBMCs from 3 different healthy donors were treated with increasing concentration of metformin (0 - 50 mM) for 18 h or 24 h, respectively. **(A, B, C)** Dead BPDCN cells (*i.e.*, CAL-1**,** GEN2.2 and BPDCN PDX cells) were determined by flow cytometry as Annexin V^+/7-AAD^+ cells (n=3). **(A)** Histogram represents the mean ± SEM of experiments of three different PDX (P224B-M2 [n=1], P178M-M1 [n=1] and P193B-M1 (n=1]). **(B, C)** Histograms represent the IC50 values of CAL-1 and GEN2.2 BPDCN cells, calculated with GraphPad Prism (version 9.0; GraphPad Software, Inc., San Diego, CA, USA). **(D)** Dead PBMCs were determined by flow cytometry Annexin V+/7-AAD+ cells (n=3). Histogram represents the mean ± SEM of experiments of three different PBMCs' healthy donors. **(E)** Histograms represent the IC50 values of PBMC cells, calculated with GraphPad Prism (version 9.0; GraphPad Software, Inc., San Diego, CA, USA).

Figure S2.

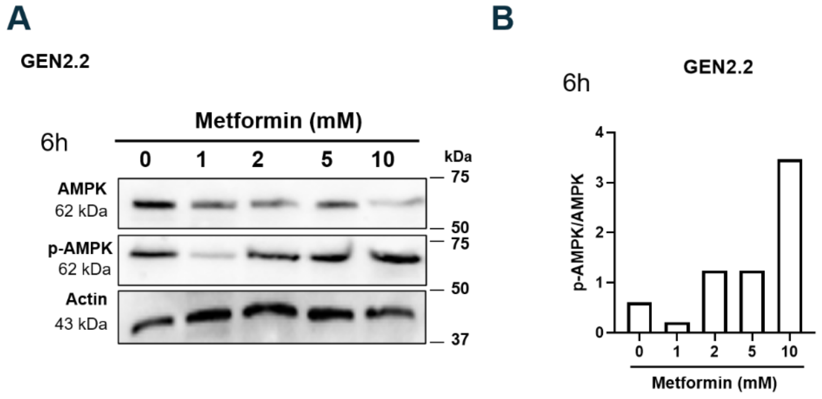

Figure S2. Metformin activates AMPK signaling pathway in blastic plasmacytoid dendritic cell neoplasm cell line GEN2.2. (A) Expression of AMPK and p-AMPK levels in GEN2.2 cell line was analyzed using Western blotting 6 hours after treatment with increasing concentrations of metformin (0 - 10 mM). **(B)** Expression was semi-quantified in GEN2.2 cells and normalized using actin and non-treated condition. Actin was used as control for protein expression. Histogram represents the p-AMPK to AMPK ratio.

Figure S3.

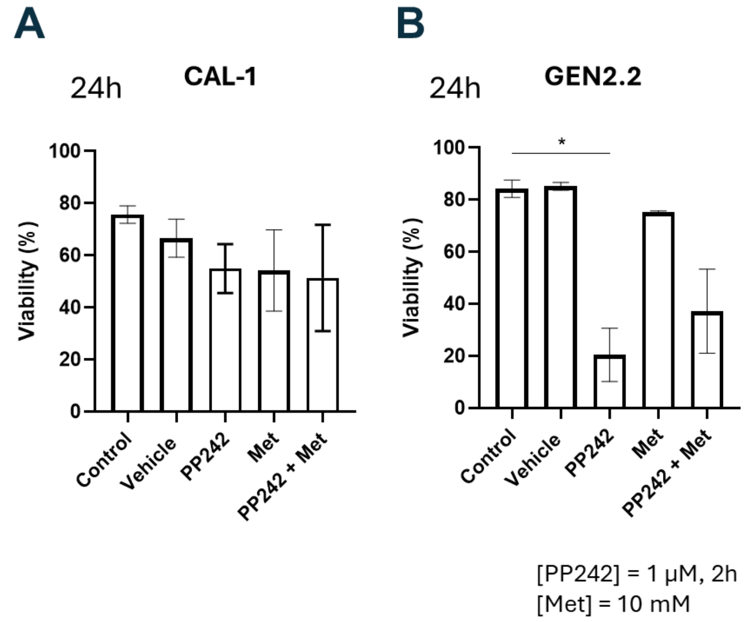

Figure S3. Metformin exerts its anti-tumor activity primarily through suppression of the mTOR axis. (A) and (B) CAL-1 and Gen2.2 cells were pre-treated with 1 µM of the mTORC1/C2 inhibitor for 2 hours, followed by incubation with 20 mM or 10 mM of metformin, respectively, for 24 hours. Viable cells were determined by flow cytometry Annexin V-/7-AAD- cells (n=3). Histogram represents the mean ± SEM of three independent experiments, **p*<0.05. The vehicle is DMSO in which PP242 has been reconstituted. Met, metformin.

Figure S4.

A

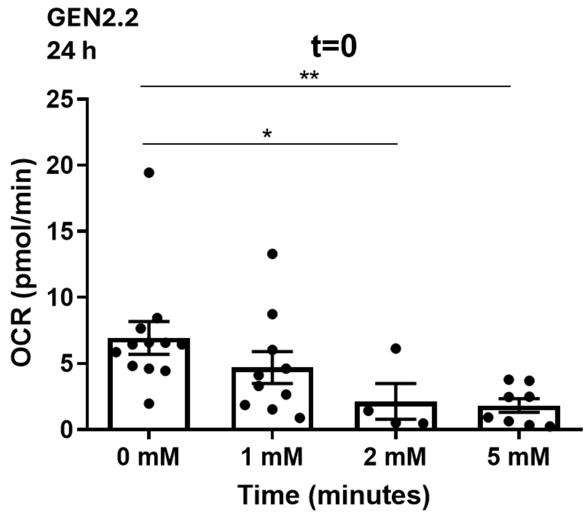

B

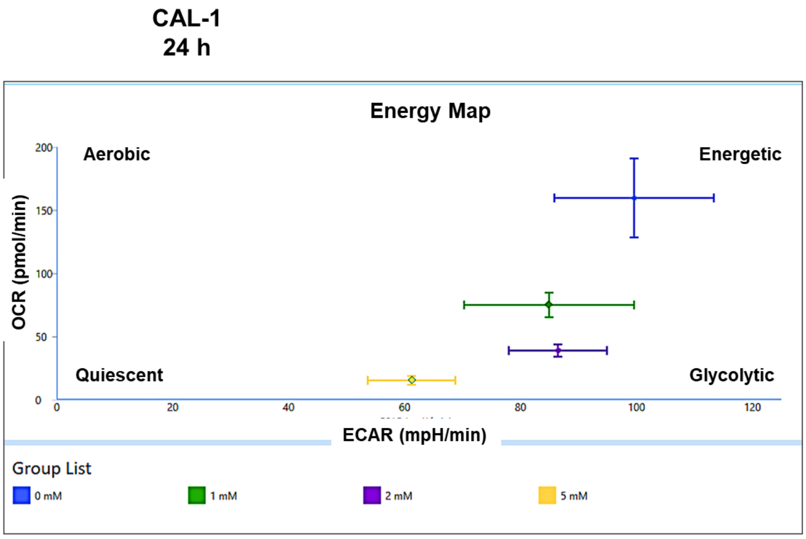

Figure S4. Metformin inhibits mitochondrial respiration in BPDCN cells. CAL-1 and GEN2.2 cells were treated with metformin (0 - 5 mM) 24 hours. **(A)** The Glycolytic Rate Assay kit was used to measure oxygen consumption rate (OCR) respiration (n=12 wells/group) using the Seahorse XF96 Analyzer. Histograms represent the mean ± SEM for 12 wells per condition and statistically significant differences are indicated *$p<0.05$, **$p<0.01$. **(B)** The Cell Mito stress test kit was used to measure the parameters of mitochondrial respiration (Energy map, n=12 wells/group). The oxygen consumption rates (OCR) profile, and the extracellular acidification rate (ECAR) were measured by an Agilent Seahorse XFe96 metabolic flux analyzer (Agilent Technologies, Centerville Road, Wilmington, USA). Additionally, the energy profile for each treatment group one representative experiment was generated using the same analyzer.

Data S1. Raw, uncropped images of the gels and immunoblot membranes.

Figure 2C

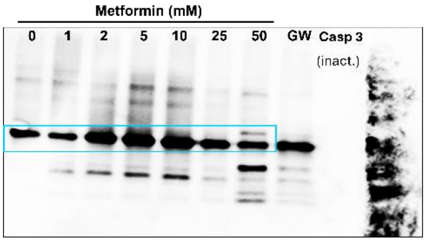

Caspase 3 inactivated

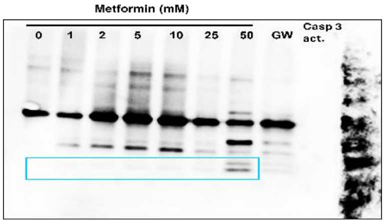

Caspase 3 activated

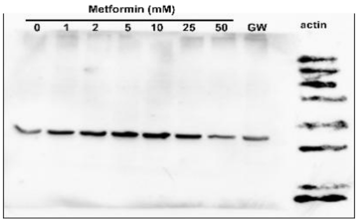

Actin

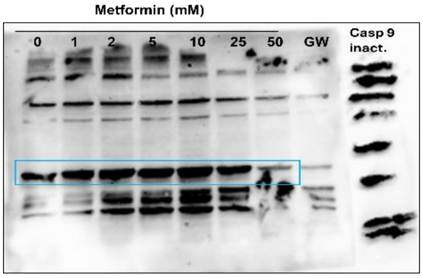

Caspase 9 inactivated

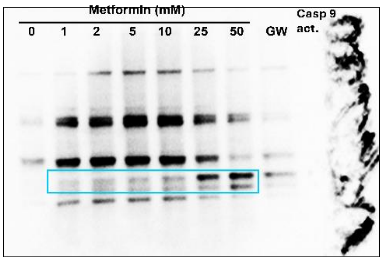

Caspase 9 activated

Figure 3A

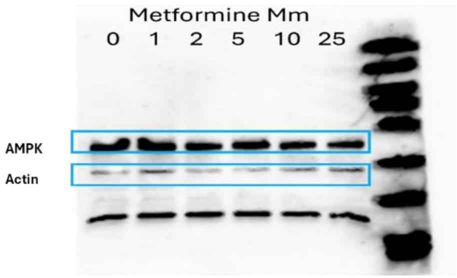

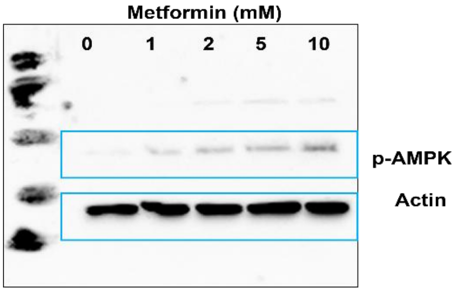

Figure 3C

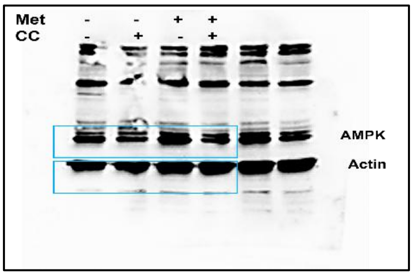

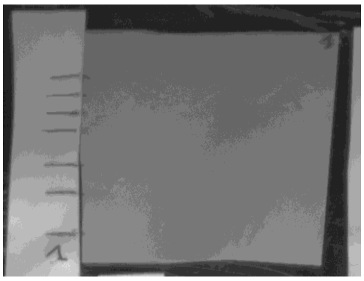

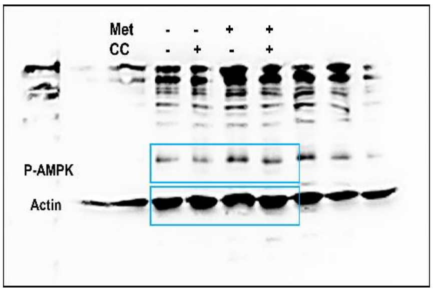

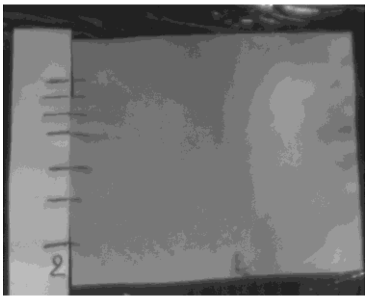

Figure S2

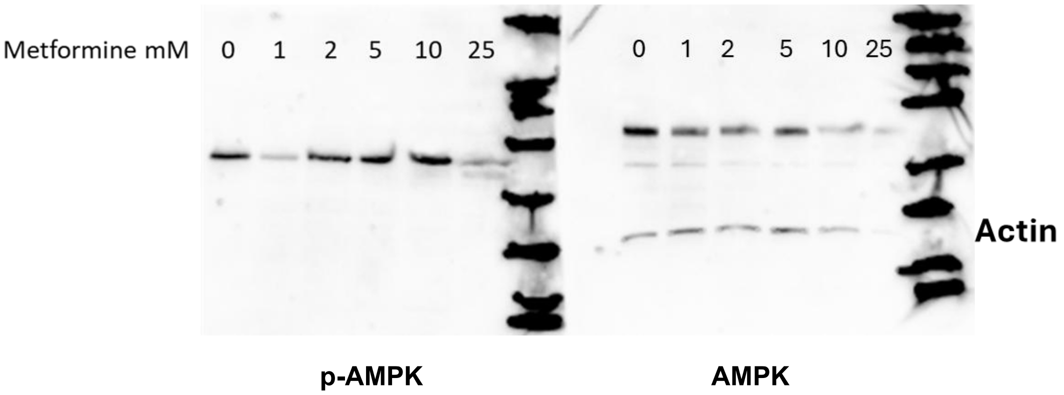
Metformine mM
0
1
2
5
10
25
0
1
2
5
10
25
Actin
p-AMPK
AMPK

Table S1. Levels of phosphorylated signaling targeted proteins in BPDCN cell lines and primary PDX cells following metformin treatment.

**BPDCN cells**	**p-Akt**	**p-mTOR**	**p-NF-κB p-65**	**p-STAT3**	**p-STAT5**
CAL-1 cell line	+****	+****	(R848)+****	(IL-3)+****	(IL-3)+****
GEN2.2 cell line	+****	-	+****	ND	ND
P224B-M2 PDX	+**	-	-	ND	ND
P178M-M1 PDX	+*	-	-	ND	ND
P193B-M1 PDX	-	+****	+**	-	+**

IL-3, IL-3 stimulation; ND, not determined; p-, phosphorylated; R848, TLR7 stimulation; +, significant inhibition; -, no effect. *, p<0.05; **, p<0.01; ****, p<0.0001.

Table S2. Phenotypic and molecular features of BPDCN used in this study

MARKERS	CAL-1 cells	GEN2.2 cells	P178M-M1	P193B-M1	P224B-M1
BPDCN-specific					
**CD4**	88%-Pos	99%-Pos	100%-Pos	100%-Pos	100%-Pos
**CD123**	90%-Pos	99%-Pos	100%-Pos	100%-Pos	100%-Pos
**HLA-DR**	100%-Pos	97%-Pos	100%-Pos	100%-Pos	100%-Pos
**cTCL1**	ND	ND	100%-Pos	100%-Pos	42%
**CD56**	96%-Pos	15%-Neg	100%-Pos	100%-Pos	100%-Pos
**CD45RA**	98%-Pos	99%-Pos	ND	ND	ND
**CD303**	94%-Pos	Pos	75%-Pos	67%-Pos	68%-Pos
**CD304**	ND	Pos	90%-Pos	65%-Pos	80%-Pos
Myeloid					
**CD33**	94%-Pos	Pos	23%-Pos	0%-Neg	ND
**CD13**	3%-Neg	4%-Neg	ND	0%-Neg	ND
**CD117**	ND	0%-Neg	ND	43%-Pos	ND
**CD36**	ND	ND	ND	100%-Pos	ND
**cMPO**	ND	ND	ND	0%-Neg	ND
T-lymphoid					
**CD2**	ND	4%-Neg	ND	ND	99%-Pos
**CD7**	3%-Neg	67%-Pos	ND	ND	2%-Neg
**cCD3**	ND	ND	ND	0%-Neg	ND
DC					
**CD11c**	23%-Pos	1%-Neg	0%-Neg	0%-Neg	ND
**CD1c**	ND	5%-Neg	0%-Neg	ND	ND
**CD141**	ND	ND	0%-Neg	21%-Pos	ND
Immature					
**cTdt**	ND	ND	ND	65%-Pos	ND
**CD38**	94%-Pos	ND	ND	ND	0%-Neg
**CD34**	0%-Neg	0%-Neg	0%-Neg	0%-Neg	0%-Neg
**NGS mutations**	ND	TET2/KRAS	TET2	NRAS	ND

c means intracytoplasmic; ND, not determined; NGS, next generation sequencing; A marker is considered as positive (Pos) when $\geq$ 20% according to Garnache-Ottou F *et al.*, Extended diagnostic criteria for plasmacytoid dendritic cell leukaemia. *Br J Haematol*. 2009. Otherwise, it is considered as negative (Neg). MPO means myeloperoxidase, Tdt, Terminal deoxynucleotidyl transferase.
